# Lipid Nanoparticle Delivery System for Normalization of Tumor Microenvironment and Tumor Vascular Structure

**DOI:** 10.34133/bmr.0144

**Published:** 2025-02-11

**Authors:** Heejin Ha, Yonghyun Choi, Na-Hyeon Kim, Jiwon Kim, Jaehee Jang, Tagbo H. R. Niepa, Masayoshi Tanaka, Hee-Young Lee, Jonghoon Choi

**Affiliations:** ^1^ School of Integrative Engineering, Chung-Ang University, Seoul 06974, Republic of Korea.; ^2^ Department of Chemical Science and Engineering, Institute of Science Tokyo, Kanagawa 226-8503, Japan.; ^3^ Department of Chemical Engineering, Kumoh National Institute of Technology, Gumi 39177, Korea.; ^4^ Department of Chemical Engineering, Carnegie Mellon University, Pittsburgh, PA, USA.; ^5^ Department of Biomedical Engineering, Carnegie Mellon University, Pittsburgh, PA, USA.; ^6^ Feynman Institute of Technology, Nanomedicine Corporation, Seoul 06974, Republic of Korea.

## Abstract

Tumors grow by receiving oxygen and nutrients from the surrounding blood vessels, leading to rapid angiogenesis. This results in functionally and structurally abnormal vasculature characterized by high permeability and irregular blood flow, causing hypoxia within the tumor microenvironment (TME). Hypoxia exacerbates the secretion of pro-angiogenic factors such as vascular endothelial growth factor (VEGF), further perpetuating abnormal vessel formation. This environment compromises the efficacy of radiotherapy, immunotherapy, and chemotherapy. In this study, we developed a pH-sensitive liposome (PSL) system, termed OD_PSL@AKB, to co-deliver oxygen (OD) and razuprotafib (AKB-9778) to tumors. This system rapidly responds to the acidic TME to alleviate hypoxia and inhibit VEGF secretion, restoring VE-cadherin expression in hypoxic endothelial cell/cancer cell cocultures. Our findings highlight the potential of OD_PSL@AKB in normalizing tumor vasculature and improving therapeutic efficacy.

## Introduction

Tumor angiogenesis is the formation of new blood vessels as the tumor grows and metastasizes. Tumors rely on the surrounding blood vessels to supply nutrients and oxygen, remove waste products, and allow metastasis to other sites. Normally, physiological angiogenesis occurs during early embryonic development, tissue regeneration, and healing. It is highly regulated and involves several steps, including the activation of vascular endothelial progenitor cells, degradation of the extracellular matrix, migration and proliferation of endothelial cells, and maturation of the vessel. In contrast, tumor neovascularization forms new blood vessels very rapidly for tumor growth, resulting in the formation of structurally and functionally abnormal tumor vessels that differ from the normal vasculature. These abnormal tumor vessels are characterized by the following features [1–4]: (a) irregular arrangement of vascular endothelial cells and an unstable vessel wall, resulting in a tortuous shape; (b) irregular blood flow due to an abnormal structure; and (c) increased permeability due to vascular immaturity and lack of mural cell adhesion. These features lead to the loss of nutrients and oxygen delivered to the tumor, causing tumor cells to secrete more proangiogenic factors. This creates a vicious cycle, and as the oxygen supply decreases, severe hypoxia develops within the tumor [5–8].

In hypoxic environments, tumors adapt by altering their cellular metabolism. Instead of generating energy through oxidative phosphorylation during hypoxia, tumor cells undergo anaerobic glycolysis in the cytoplasm to generate adenosine triphosphate (ATP) [9,10]. This leads to the excessive accumulation of lactic acid, which acidifies the tumor microenvironment (TME). In addition, tumors have increased expression levels of the hypoxia-inducible factor-1α (*HIF-1α*) gene to adapt to low oxygen concentrations [11,12]. Normally, HIF-1α is hydroxylated by the prolyl hydroxylase domain (PHD) in the presence of normal oxygen levels. Hydroxylated HIF-1α is recognized by the von Hippel–Lindau (VHL) protein and is degraded by the proteasome. However, under conditions of low oxygen concentration, it is reversely stabilized and acts as a transcription factor for hypoxia-related factors. One of these factors is vascular endothelial growth factor (VEGF), which is a pro-angiogenic factor and one of the most influential factors in promoting angiogenesis. Additionally, HIF regulates the expression of MMPs (matrix metalloproteinases), integrins, CXCR4 (C-X-C chemokine receptor type 4), and others to enhance the ability of tumor cells to survive and invade other tissues [13–16]. This hypoxic TME promotes the deposition of immunosuppressive cells [e.g., regulatory T cells and myeloid-derived suppressor cells (MDSCs)] and increases the expression of immunosuppressive components (PD-L1), which inhibit the function of immune cells, such as T cells and natural killer cells, thereby enabling immune evasion. In addition, increased expression of multidrug resistance protein (MDR1) prevents anticancer drugs from entering the cell. Coupled with unstable vasculature, drug accumulation within the tumor is reduced, leading to anticancer drug resistance [17–19]. Overcoming abnormal tumor vasculature and hypoxia is crucial for the treatment of tumors, as these constitute major barriers to the therapeutic effectiveness of radiotherapy, immunotherapy, and chemotherapy.

To overcome the increased hypoxia caused by abnormal tumor vasculature and the deterioration of the TME, many therapeutic strategies targeting the tumor vasculature have been implemented. Most commonly, anti-angiogenic strategies that inhibit tumors from producing blood vessels focus on inhibiting VEGF and blocking its receptors. For example, the monoclonal antibody bevacizumab, which targets VEGF, prevents it from binding to its receptor (VEGFR). Sorafenib and sunitinib are multikinase inhibitors that block neovascularization by inhibiting VEGFR and the platelet-derived growth factor receptor (PDGFR), and apatinib is a tyrosine kinase inhibitor that selectively inhibits VEGFR-2. They aim to interfere with the progression of diseases, such as tumors, by blocking abnormal blood vessel formation. Vascular disrupting agents (VDAs), which destroy already formed tumor blood vessels rather than inhibiting the formation of new blood vessels, mainly interfere with the proliferation and survival of tumor cells by damaging the structure of tumor blood vessel endothelial cells and destroying blood vessel walls. One such example is combretastatin. Embolization is a local treatment method that uses particulates, liquids, and coils through a catheter to block blood flow to a tumor [20–23]. However, these treatment strategies have limitations in that they delay tumor growth, but do not induce tumor death. In addition, because they block oxygen and nutrients from entering the tumor, tumor hypoxia is exacerbated, which increases the likelihood of tumor metastasis. Therefore, “vascular normalization” strategies have recently focused on stabilizing the already abnormally formed tumor blood vessels, rather than inhibiting the formation of tumor neovessels, to maximize the efficiency of oxygen supply to and drug accumulation in the tumor [24–27].

Various signal transduction pathways may be involved in normalizing the tumor vasculature. The Tie-2 receptor is an important protein present in vascular endothelial cells. It regulates signaling pathways involved in vascular stability and development. Inducing activation of the Tie-2 pathway is important for controlling the normalization of abnormal tumor blood vessels. There are 5 main components of the Tie-2 pathway: Tie-1 and Tie-2 receptors, angiopoietin-1 (ANG1), angiopoietin-2 (ANG2), and vascular endothelial tyrosine phosphatase (VE-PTP) receptors. ANG1 and ANG2 bind to the Tie-2 receptor. ANG1 is mainly expressed in smooth muscle cells, perivascular cells, and fibroblasts. When ANG1 binds, it activates the Tie-2 receptor, which is involved in endothelial cell stabilization and vascular maturation. Conversely, ANG2 binding interferes with the Tie-2 receptor, reducing vascular stability and enhancing the action of VEGF to increase vascular permeability, thereby supporting the abnormal growth of tumor vessels. In addition, VE-PTP is a transmembrane receptor tyrosine phosphatase that dephosphorylates the Tie-2 receptor, leading to down-regulation of the Tie-2 pathway. Inhibition of VE-PTP may promote signaling pathway activation by maintaining the Tie-2 receptor in an actively phosphorylated state [28,29].

**Fig. 1.**
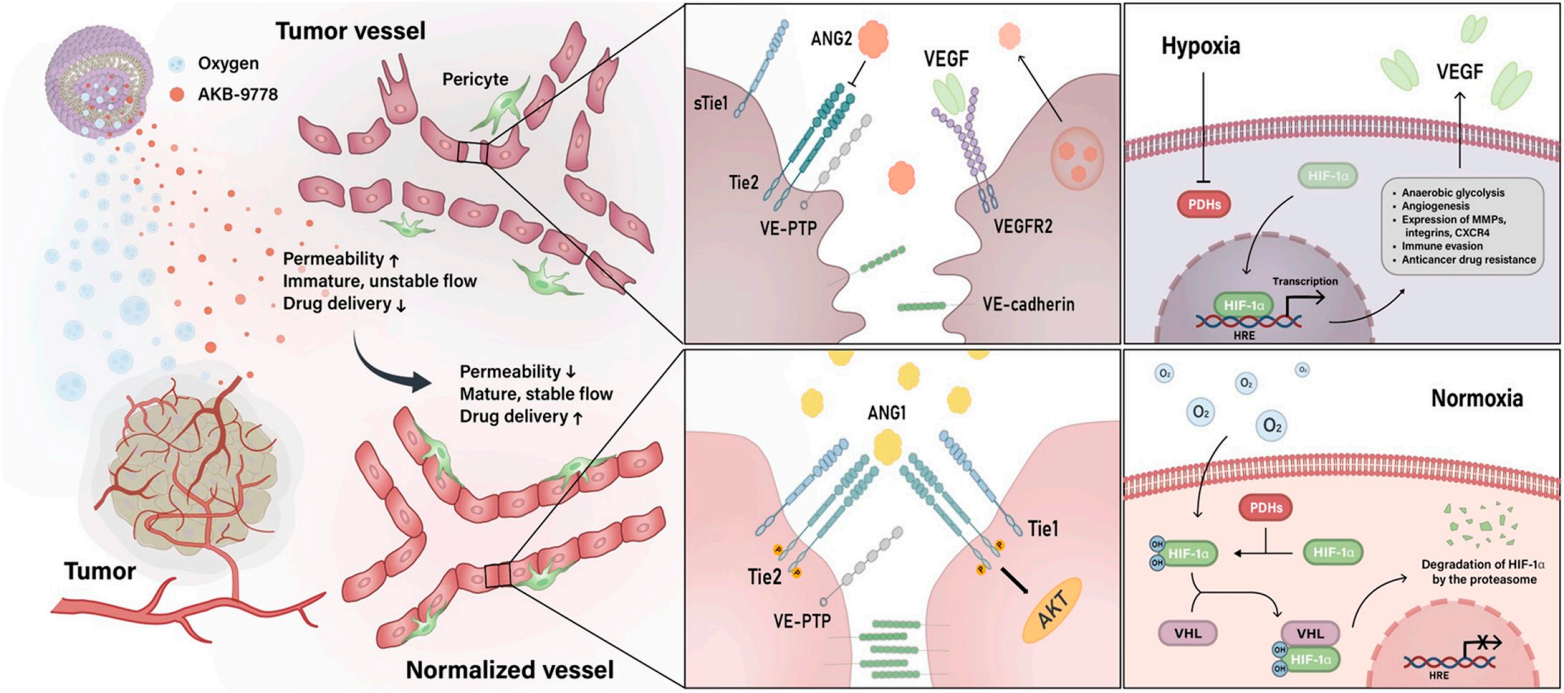
Schematic illustration of TME modulation and tumor vascular normalization via OD_PSL@AKB delivery. OD_PSL@AKB, loaded with oxygen and AKB-9778, is a pH-sensitive lipid nanoparticle that can rapidly release its cargo in response to the acidic TME. This relieves the hypoxic environment by delivering oxygen, which in turn reduces the production of VEGF by inhibiting HIF-1α. In addition, AKB-9778, a VE-PTP-targeting small-molecule inhibitor, achieves vascular normalization through vascular maturation and stabilization by activating the Tie-2 pathway. This reduces the permeability of tumor vessels and allows normal blood flow, which may enhance drug uptake into the tumor.

Razuprotafib (AKB-9778) is a small-molecule inhibitor that selectively targets VE-PTP. It is primarily used to treat diabetic retinopathy, macular degeneration, retinal vein occlusion, and other vascular complications associated with abnormal blood vessel growth. By inhibiting the action of VE-PTP to dephosphorylate the Tie-2 receptor, AKB-9778 induces the activation of the Tie-2 signaling pathway, thereby stabilizing blood vessels and enhancing endothelial function. This has the potential to effectively promote tumor vascular normalization and improve tumor vascular perfusion, thereby reducing hypoxia [30,31]. Oxygen delivery to the TME plays a key role in alleviating hypoxia. In our previous study, we developed oxygen nanosomes capable of delivering oxygen and found that they substantially reduced the expression level of HIF-1α. Furthermore, immunonanosomes conjugated with anti-CD73 antibody on the surface of the particles effectively induced antitumor immune activity by reducing the activity of CD73 and adenosine, in addition to alleviating hypoxia [32,33]. One study examined how liposome rigidity influences oxygen delivery, revealing that incorporating cholesterol and polymers enhanced the rigidity of the bilayer membrane, thereby impacting the oxygen release rate [34]. Based on this, in this study, we synthesized a pH-sensitive liposome (PSL) that rapidly responds to the TME, which has a relatively acidic environment. In an acidic environment, it uses the principle of interaction between dioleoyl phosphatidylethanolamine (DOPE) and cholesteryl hemisuccinate (CHEMS). We found that, at neutral pH, CHEMS remained in an ionized form, allowing it to interact with DOPE and maintain a stable lamellar liposome structure. However, at acidic pH, CHEMS became protonated, which changed its interaction with DOPE and promoted transition to the hexagonal phase (HII phase), reducing the stability of the liposome structure and causing it to become unstable and release the loaded cargo [35–38]. Finally, we developed OD_PSL@AKB, which could co-deliver oxygen and AKB-9778 inside the PSL (Fig. 1). We confirmed the decreased expression level of VEGF and the restoration of VE-cadherin expression in vascular endothelial cells using the hypoxic human umbilical vein endothelial cell (HUVEC)/cancer cell coculture system. Therefore, OD_PSL@AKB has the potential to relieve hypoxia in the TME and normalize abnormal tumor vasculature. Promoting normoxia with this method can enhance the efficacy of therapeutic agents and inhibit tumor progression, highlighting the importance of OD_PSL@AKB in cancer treatment.

## Materials and Methods

### Materials

18:1 (Δ9-Cis) PE (DOPE; 1,2-dioleoyl-sn-glycero-3-phosphoethanolamine), CHEMS, DSPC (18:0 PC) (1,2-distearoyl-sn-glycero-3-phosphocholine), and 18:0 PEG2000 PE (1,2-distearoyl-sn-glycero-3-phosphoethanolamine-N-[methoxy (polyethylene glycol)-2000], ammonium salt) were purchased from Avanti Polar Lipid Inc. (Alabaster, AL, USA). Pure oxygen gas (99.5%) was purchased from GT Korea (Seoul, Korea), and syringe filters were purchased from Whatman (Maidstone, UK). SpectraPor 12- to 14-kDa dialysis bags, acetonitrile (ACN) for high-performance liquid chromatography (HPLC), trifluoroacetic acid (TFA), Triton X-100, and bovine serum albumin (BSA) were purchased from Sigma-Aldrich (St. Louis, MO, USA). The rabbit anti-VE-cadherin polyclonal antibody and fluorescein isothiocyanate (FITC) goat anti-rabbit IgG (H+L) were purchased from ABclonal Inc. (Woburn, MA, USA). The VEGF enzyme-linked immunosorbent assay (ELISA) kit was purchased from BioLegend (San Diego, CA, USA). The modular incubator chamber was purchased from Billup Rothenberg (San Diego, CA, USA). The gas for hypoxic cell culture (1 cmol/mol O_2_, 4.99 cmol/mol CO_2_, and N_2_ balanced) was customized by Deokyanggas Co. Ltd. (Seoul, Korea). Mounting medium with 4′,6-diamidino-2-phenylindole (DAPI) was purchased from Vectorlabs (Newark, CA, USA). Matrigel, collagen I, and rat tails were purchased from Corning (Corning, NY, USA). Human VEGF165 protein (HEK293) and razuprotafib (AKB-9778) were purchased from MedChemExpress (Monmouth Junction, NJ, USA).

### Preparation of PSLs

To prepare PSLs, a lipid solution was first prepared by mixing DOPE, CHEMS, DSPC, and 18:0 PEG2000 PE dissolved in chloroform in a glass vial in a molar ratio of 37.5:20:37.5:5. The chloroform was evaporated in a 70 °C oven for 2 h, and when it had completely evaporated and a white thin film was formed, the sample was resuspended with 10 ml of Dulbecco’s phosphate-buffered saline (DPBS). The lipid solution was then dispersed using a bath sonicator (100 W) at a temperature above the transition temperature (i.e., 55 °C) for 10 min. The lipid solution was then dispersed once more using a tip sonicator (on 5 s/off 5 s, 5 min, room temperature, 20% amplitude). Finally, a 1.0-μm polytetrafluoroethylene (PTFE) filter was used for primary removal of impurities. PSL@AKB and AKB-9778 were mixed and dried during the lipid-drying step, following identical procedures. PSL@AKB was obtained by separating it from the free drug using an ultracentrifuge (1000,000*g*, 1 h, 4 °C) rather than a 1.0-μm PTFE filter. Oxygen-loaded OD_PSLs were obtained by drying the lipid, resuspending it in DPBS, dispersing it in a bath sonicator above the transition temperature, and saturating the lipid solution with oxygen gas for 3 min. They were then dispersed using a tip sonicator and synthesized by adding oxygen gas during the dispersion process. Finally, OD_PSL@AKB was obtained by simultaneously performing these 2 processes. All particles were stored at 4 °C until used in the experiments.

### Characterization

#### Dynamic light scattering

The sizes and surface charges of the synthesized particles were measured using a Zetasizer Pro instrument (Malvern Instruments, Malvern, UK). The samples were diluted to the appropriate concentrations in distilled water and measured using a dedicated zetasizer cell. All samples were measured at least in triplicate.

#### Nanoparticle tracking analysis

The concentration of each particle was measured using a NanoSight LM10 NTA instrument (Malvern Instruments). All samples were diluted to the appropriate concentration, and the final concentration was calculated by applying the dilution factor to the measured concentration.

#### Field-emission transmission electron microscopy

Transmission electron microscopy (TEM) analysis was used to confirm the morphology and membrane modification of the synthesized particles. Each sample was placed on a carbon-coated nickel mesh grid for several minutes, stained with 2% uranyl acetate, and dried. Each image was analyzed by FE-TEM (JEM-F200; JEOL Ltd., Tokyo, Japan) at a voltage of 80 kV. The measured particles were incubated in different pH environments of DPBS (pH 7.4, 6.5, and 5.0) for >12 h before measurement.

#### Small-angle x-ray scattering

To confirm that the synthesized particles were liposomes, they were analyzed using small-angle x-ray scattering (SAXS) (4C SAXS II beamline; Pohang Accelerator Laboratory, Pohang, South Korea). X-rays with an energy of 16.9 keV were used, and the scattering patterns were analyzed using a MAR charge-coupled device detector (MAR-CCD) area detector. For the measurements, each sample was injected into a 1.5-mm quartz capillary cell, and the distance between the sample and detector was set to 1 and 4 m, respectively, to obtain data in the range of 0.007 to 0.3 Å^−1^. The magnitude of the scattering vector is *q* = (4π/λ) sin θ, where 2θ is the scattering angle and λ is the wavelength of the x-ray beam.

### Dissolved oxygen concentration and oxygen release using dissolved oxygen meter

An optical dissolved oxygen (DO) meter (YSI, Yellow Springs, OH, USA) was used to measure the oxygen concentration in the liposomes. The sample was measured by removing impurities through a 1.0-μm PTFE filter immediately after synthesis and then taking 5 ml of the sample. The samples were sealed with parafilm and measured in a closed environment.

The oxygen release tendencies of the particles were measured using a DO meter. Initially, 18 ml of DPBS was placed in a 25-ml beaker, and argon gas was bubbled through the buffer until the DO concentration dropped below 0.2 mg/l. To prevent atmospheric oxygen from affecting the measurements, the beaker was sealed with parafilm to maintain a closed system. Subsequently, 2 ml of the sample was added, followed by exposure to argon gas for 5 min. The oxygen concentration was recorded every 30 s for 40 min.

### Encapsulation efficiency

Free drugs were unloaded from PSL@AKB and OD_PSL@AKB through ultracentrifugation at 100,000*g* for 1 h at 4 °C. To determine the concentration of encapsulated AKB-9778, supernatants containing the free drugs were analyzed using an HPLC 1260 Infinity II system (Agilent, Santa Clara, CA, USA). A Poreshell 120 EC-C18 column (Agilent) was used, the mobile phase was buffer A (distilled water with 0.1% TFA) and buffer B (ACN with 0.1% TFA), the detection wavelength was 320 nm, the flow rate was 1 ml/min, and the temperature was 30 °C. The calibration curve was calculated as a linear regression equation using AKB-9778 concentrations, and the amount of free drug was quantified by substituting the corresponding equations. Encapsulation efficiency (EE%) was calculated using the following formula:

$$\begin{array}{c} \mathrm{EE}\;\left(\%\right)=\\ \frac{\text{Total amount of drug}-\text{unloaded drug amount}}{\text{Total amount of drug}}\times 100. \end{array}$$

### Drug release test

In vitro drug release studies were performed using the dialysis bag method. For PSL@AKB, 2 ml of the sample was transferred to a dialysis membrane bag (molecular weight cutoff, 12,000 to 14,000 Da; Spectrum Spectra/Por 3 RC Dialysis Membrane Tubing, USA), and then DPBS buffer containing 10 ml of 30% ethanol was used as an external buffer. In addition, an external buffer was prepared at pH 7.4, 6.5, and 5.0, and the reaction was allowed to proceed under these 3 conditions. Thereafter, the tube containing the bag was placed in an orbital shaker at 37 °C. Incubation was performed, and 1 ml of each sample was recovered at regular time intervals and the same volume of fresh external buffer was added. The concentration of AKB-9778 in the recovered sample was measured and analyzed under the HPLC conditions described in the “Encapsulation efficiency” section.

### Cell culture and hypoxic culture

HeLa (a cervical cancer cell line) and Panc-1 (a pancreatic cancer cell line) cells were purchased from the Korea Cell Link Bank (Seoul, Korea), while MDA-MB-231 cells (a breast cancer cell line) and HUVECs were purchased from the American Type Culture Collection (VA, USA). Cancer cell lines PANC-1, HeLa, and MDA-MB-231 were cultured in high-glucose Dulbecco’s modified Eagle’s medium (DMEM; WELGENE, Korea). HUVECs were cultured using EGM Endothelial Cell Growth Medium BulletKit (Lonza, Basel, Switzerland). The medium used was 10% or 2% fetal bovine serum (FBS; Gibco, Waltham, MA, USA), 1% antibiotics–antimycotics (A/A; Hyclone, Logan, UT, USA) was added, and the cells were cultured at 37 °C under 5% CO_2_. For hypoxic cell culture, cells were placed in an incubator chamber purged with a mixture of 1% O_2_/4.99% CO_2_/94.01% N_2_ gas, and the chamber was placed in an incubator at 37 °C and 5% CO_2_ and incubated for 24 to 48 h.

### In vitro cell cytotoxicity

The Cell Counting Kit-8 (CCK-8) assay was performed to evaluate the toxicity of the synthesized PSLs, PSL@AKB, OD_PSL, and OD_PSL@AKB. Cells (100 μl) were seeded in 96-well plates at a concentration of 1 × 10^4^ cells/well. They were incubated for 24 h in an incubator at 37 °C and 5% CO_2_. After 24 h, the existing medium was removed, and each particle was diluted in fresh medium to the appropriate concentration and added to each well. One percent Triton X-100 was used as a positive control. After 24 h of sample treatment, the CCK-8 solution was replaced with medium containing 1:10 (v/v) and incubated for 2 h. The absorbance was measured at 450 nm using a microplate reader (BioTek, Winooski, VT, USA). The measurement results were compared by calculating cell viability relative to the untreated negative control.

### Tube formation assay

A tube formation assay was performed to evaluate the maintenance and stability of tubular structure formation. A pre-cooled 24-well plate was coated with 200 μl of Matrigel (Corning) and incubated at 37 °C for polymerization. HUVECs were seeded at a density of 6 × 10^4^ cells/ml per well. VEGF (10 ng/ml) treatment was used as a positive control, and cells were treated with free AKB-9778 and PSL@AKB at a concentration of 10 μM. After incubation for 6, 12, and 24 h, endothelial tube formation was evaluated under a light microscope. For imaging analysis, the number of master junctions and the total segment length were quantified using ImageJ software (National Institutes of Health, Bethesda, MD, USA) using the “Angiogenesis Analyzer” tool.

### Permeability test

A permeability test was performed using a transwell chamber to evaluate the permeability of vascular endothelial cells. First, the membrane of the transwell chamber was coated with collagen I at a concentration of 50 μg/ml. HUVECs were seeded on the insert in the upper chamber of the transwell apparatus [24 wells, 0.4-μm pore size, polyethylene terephthalate (PET) membrane] at a concentration of 5 × 10^4^ cells/well, and 600 μl of fresh media was injected into the lower chamber. After that, it was incubated in an incubator at 37 °C and 5% CO_2_ until confluency reached 100%. When 100% confluence was achieved, the lower chamber was treated with 10 ng/ml VEGF and incubated for 1 h. Subsequently, free AKB and PSL@AKB were added to the lower chamber to a concentration of 10 μM and incubated again for 30 min. Finally, FITC–dextran was added to the upper chamber at a concentration of 4 mg/ml and 100 μl of media was recovered from the lower chamber after 24 and 48 h. The recovered samples were diluted to 1:10, and the permeability was calculated by measuring the amount of FITC–dextran detected at a wavelength of excitation 488 nm/emission 520 nm using a microplate reader.

### VE-cadherin immunofluorescence

To confirm the stabilization of the barrier function of vascular endothelial cells, the expression of VE-cadherin, a cell–cell attachment molecule, was confirmed using immunofluorescence. First, HUVECs were seeded at a concentration of 8 × 10^4^ cells/ml on a 12-well plate with a coverslip and incubated in an incubator at 37 °C under 5% CO_2_ until confluency reached 80 to 90%. Thereafter, the medium was removed and replaced with fresh medium or medium containing VEGF (10 ng/ml), and the cells were incubated for 1 h. Subsequently, the cells were treated with free AKB or PSL@AKB at a concentration of 10 μM for 30 min to induce the recovery of weakened cell junctions. After treatment, the cells were fixed with 2% paraformaldehyde for 30 min and washed once with 1× PBS. After fixation, the cells were washed 2 to 3 times with 1× PBS and treated with 1% BSA for 1 h to prevent nonspecific defects. The 1% BSA solution was removed, and a rabbit anti-VE-cadherin polyclonal primary antibody was added at a dilution of 1:200 and incubated overnight at 4 °C. The next day, the solution was removed and the cells were washed 3 times with 1× PBS and incubated at room temperature for 1 h with FITC-conjugated goat anti-rabbit IgG (H+L) diluted to 1:500. In the middle of the incubation period, rhodamine phalloidin diluted to 1:400 was added and actin staining was performed. Finally, the sections were washed 3 times with 1× PBS and attached to a microscope slide with mounting medium containing DAPI. Fluorescence imaging was performed using a STELLARIS 5 confocal microscope (LEICA, Wetzlar, Germany).

### Coculture with cancer cell and cytokine measurement

HUVECs were seeded at a concentration of 1 × 10^5^ cells/ml in a 24-well plate bottom and incubated in an incubator at 37 °C under 5% CO_2_ until confluency reached 80 to 90%. After incubation, the medium was removed and replaced with basal medium that did not contain supplements. Subsequently, a transwell insert was inserted into each well, and the 3 cancer cell lines—PANC-1, HeLa, and MDA-MB-231 cells—were seeded at a density of 1 × 10^4^ cells/well. The cells were cultured for 24 and 48 h under normal and hypoxic conditions, respectively. Subsequently, the lower chamber samples and Oxy-DPBS (3-min bubbling) were replaced with fresh basal medium, and the insert was replaced with fresh medium and incubated for 3 h. After incubation, the medium in the lower chamber was recovered, and the residual cells were centrifuged. The isolated supernatant was analyzed using VEGF ELISA. All analyses were performed according to the manufacturer’s instructions.

### Statistics

One-way analysis of variance was performed using GraphPad Prism 7.0 for Windows (GraphPad Software Inc., La Jolla, CA, USA). Each experiment was performed in triplicate, and error bars indicate SDs. Nonsignificant values are represented as ns, whereas *, **, ***, and **** indicate *P* values < 0.0332, 0.0021, 0.0002, and 0.0001, respectively.

## Results and Discussion

### Characterization of PSLs

**Fig. 2.**
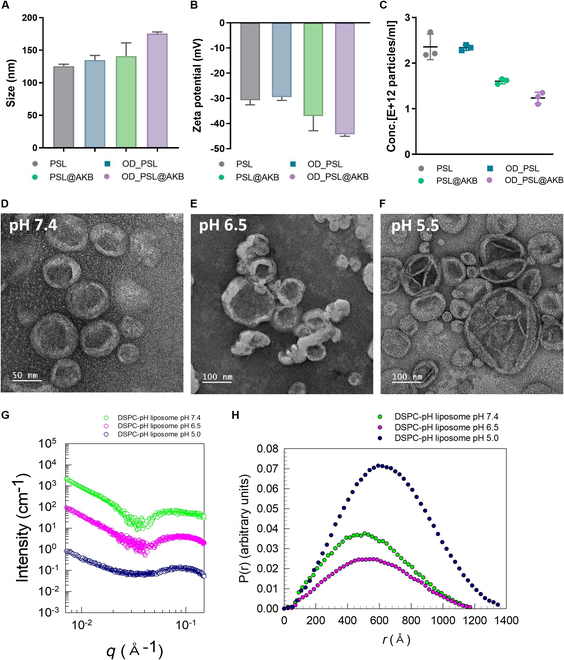
Characterization of PSLs. (A) DLS size data. (B) DLS zeta potential data. (C) NTA particle concentration data. (D to F) PSL TEM image data in pH 7.4, 6.5, and 5.5 buffer. (G) SAXS data of PSL. (H) IFT analysis of PSL in pH 7.4, 6.5, and 5.5.

In this study, we synthesized PSLs that are sensitive to relatively low pH and can rapidly release their cargo. The particles were then loaded with oxygen and razuprotafib (AKB-9778). Particles containing only oxygen were named OD_PSL, those containing only the drug were named PSL@AKB, and those containing both oxygen and the drug were named OD_PSL@AKB. Each synthesized particle was characterized. Dynamic light scattering (DLS) measurements of the PSLs showed that the average size of the nanoparticles was 125.5 nm, followed by OD_PSL, PSL@AKB, and OD_PSL@AKB at 134.9, 141.1, and 175.6 nm, respectively (Fig. 2A). The surface potential of nanoparticles is one of the factors that determine their stability. In general, a value of <−30 mV or >30 mV provides sufficient interparticle repulsion and maintains a stable particle suspension [39,40]. The zeta potential values of all synthesized particles were negative, with values between −30 and −40 mV, confirming that all particle shapes were stably synthesized (Fig. 2B). The concentration of the synthesized particles was measured using nanoparticle tracking analysis (NTA). The particle concentration of the PSLs was approximately 2.36 × 10^12^ particles/ml and OD_PSL was approximately 2.34 × 10^12^ particles/ml, while the particle concentrations of PSL@AKB and OD_PSL@AKB were 1.6 × 10^12^ particles/ml and 1.22 × 10^12^ particles/ml, respectively (Fig. 2C).

The synthesized PSLs were subjected to FE-TEM and SAXS analyses to determine the changes in particle morphology and shell structure in environments with different pH values. The synthesized PSLs were found to have a uniform size in the form of spherical liposomes at pH 7.4. At pH 6.5, the particles were slightly distorted but maintained their spherical shape. However, at pH 5.5, unlike the previous 2 pH environments, the size of the particles varied and neighboring particles were fused together or the membrane morphology was unstable (Fig. 2D to F). Furthermore, SAXS analysis was utilized to characterize PSLs and SAXS spectra (intensity *I* versus wave-vector *q*) for the liposomes at pH 7.4, pH 6.5, and pH 5.5 (Fig. 2G). Here, the experimental data were rescaled by factors of 10 for clarity purpose. Typically, liposomes with phospholipid bilayers are characterized by intensity decay slope of −2 at low *q* on the log–log plot. The results of the analysis for all samples showed a −2 slope of intensity at low *q*, indicative of liposomes with bilayered structures. In addition, the shape and size of PSLs were estimated by using the indirect Fourier transformation (IFT) method. Here, the incoherent background is determined by the asymptotic slope of a Porod plot (*Iq*^4^ versus *q*^4^) at high *q* and then the scattering intensity *Iq* with incoherent background subtracted is Fourier-transformed to find the pair distance distribution function [*p*(*r*)] in real space [41]. *p*(*r*) curves of all samples have a single broad peak, indicating the existence of spherical liposomes. In addition, the sizes of liposomes at pH 7.4, pH 6.5, and pH 5.5, estimated from the point at which *p*(*r*) touches the *r* axis, were 118, 119, and 139 nm, respectively. The abrupt increased size of the liposomes at pH 5.5 can be attributed to the fusion of the liposomes as confirmed by FE-TEM images (Fig. 2H).

### Oxygen and AKB-9778 in vitro release study

**Fig. 3.**
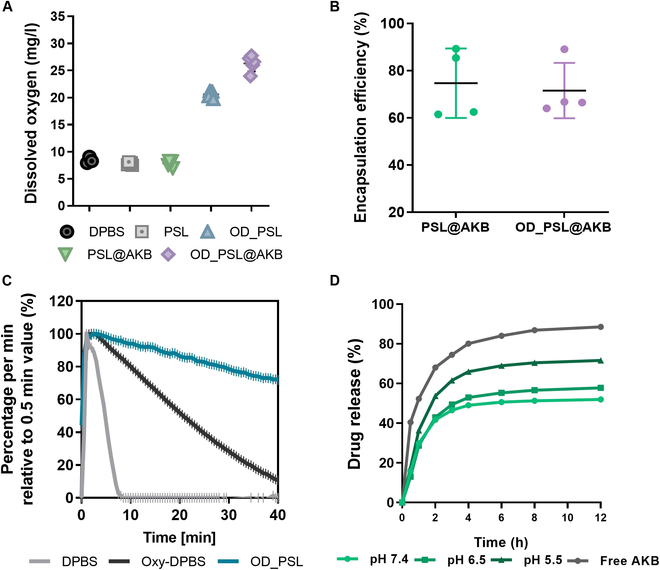
Oxygen and AKB-9778 in vitro release study. (A) DO levels in DPBS buffer, PSLs, OD_PSL, PSL@AKB, and OD_PSL@AKB measured using an optical DO meter. (B) The encapsulation efficiency (%) of AKB_9778 in PSL@AKB and OD_PSL@AKB was calculated by measuring the concentration of removed free AKB-9778. Analysis was conducted using HPLC. (C) Oxygen release tendency from DPBS, Oxy-DPBS, and OD_PSL using a DO meter. Normalization was performed using the highest recorded oxygen measurement as the reference point, during a total duration of 40 min. (D) AKB-9778 release tendency under different pH conditions of free AKB-9778 and PSL@AKB. Drug release was accelerated at lower pH levels, confirming the pH-sensitive release properties.

An optical DO meter was used to measure the oxygen concentration in each particle. DPBS buffer without oxygen had an oxygen concentration of approximately 8.41 mg/l. In comparison, the oxygen concentration in OD_PSL was found to be approximately 20.60 mg/l, and OD_PSL@AKB had an oxygen concentration of approximately 26.31 mg/l. We also confirmed that PSL and PSL@AKB synthesized without oxygen had similar oxygen concentrations as the DPBS buffer (Fig. 3A). Having confirmed that OD_PSL and OD_PSL@AKB contained high levels of oxygen, we further evaluated the ability of the particles to release the oxygen contained in their cores. To evaluate oxygen release, we used argon gas to remove all DO in the DPBS buffer until the oxygen concentration was less than 0.2 mg/l. The samples were then subjected to argon gas treatment for 5 min after injection and kept in a closed system to compare the oxygen release tendencies. For comparison, the measured data were normalized to the maximum oxygen concentration of each sample. The oxygen-free DPBS was rapidly consumed by argon gas treatment after sample injection, resulting in an oxygen concentration of 0 mg/l after 8 min. OD_PSL maintained an oxygen concentration of approximately 7% to 80% after 40 min under the same conditions. For accurate comparison, we prepared an Oxy-DPBS buffer by saturating the DPBS buffer with excess oxygen for 3 min to confirm the same oxygen release tendency. We found that the Oxy-DPBS buffer maintained an oxygen concentration of approximately 10% after 40 min. Although the oxygen concentration decreased slowly compared to that in DPBS buffer without oxygen, it was not maintained and steadily decreased compared to that in OD_PSL. This confirms that, unlike the Oxy-DPBS buffer, OD_PSL can maintain a certain level of oxygen concentration because the carrier stably retains oxygen in the core while gradually releasing oxygen (Fig. 3C).

The encapsulation efficiency (EE%) of AKB-9778 in the particles was calculated using HPLC. After particle separation by ultracentrifugation, the concentration of free AKB-9778 present in the supernatant was measured and the EE% value was calculated relative to the total amount of drug loaded. The drug entrapment efficiencies of PSL@AKB and OD_PSL@AKB were 74.68% and 71.58%, respectively (Fig. 3B). To determine the pH sensitivity of the synthesized PSLs, we evaluated drug release in different pH environments in vitro. For free AKB-9778 without particles, more than 60% of the drug was released from the dialysis bag after 2 h, and approximately 90% of the drug was released after 12 h. In contrast, PSL@AKB showed pH-dependent drug release at 2 h, with approximately 41.74% of the drug released in the pH 7.4 environment, 42.96% in the pH 6.5 environment, and 53.68% in the pH 5.5 environment. After 12 h, drug release was approximately 51.97% at pH 7.4, 57.82% at pH 6.5, and 71.66% at pH 5.5. Overall, PSL@AKB showed a tendency for controlled release compared to free AKB-9978 at all pH levels, demonstrating that increased drug release was possible in response to lower pH environments (Fig. 3D).

### Cell cytotoxicity test

**Fig. 4.**
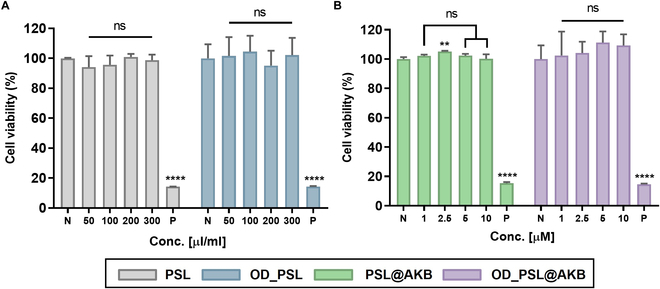
Cytotoxicity test. Cytotoxicity test of PSL, OD_PSL, PSL@AKB, and OD_PSL@AKB to HUVECs (vascular endothelial cells). (A) Data for PSL and OD_PSL, obtained through sample processing at a volume ratio (% v/v). (B) Data for PSL@AKB and OD_PSL@AKB, acquired through processing at the μM ratio of AKB-9778. Nonsignificant values are represented as ns, while ** and **** indicate *P* values < 0.0021 and 0.0001, respectively.

To determine whether the synthesized particles were toxic to the vascular endothelial cells, a CCK-8 assay was performed. In general, the phospholipids that make up liposomes are well known for their biocompatibility and are approved by the US Food and Drug Administration (FDA) as delivery vehicles [42,43]. Previous studies have demonstrated that oxygen nanosomes, which contain oxygen in liposomes, are not cytotoxic to normal cells [34,44]. Consistent with previous studies, we treated HUVECs with PSLs and OD_PSL at various concentrations (v/v%) and found that they were not toxic, with cell viability similar to that of the negative group at all concentrations. Similarly, drug-loaded PSL@AKB and OD_PSL@AKB also showed no toxicity at all concentrations (μM) treated (Fig. 4A and B).

### Stabilization of tube formation and permeability of HUVECs

**Fig. 5.**
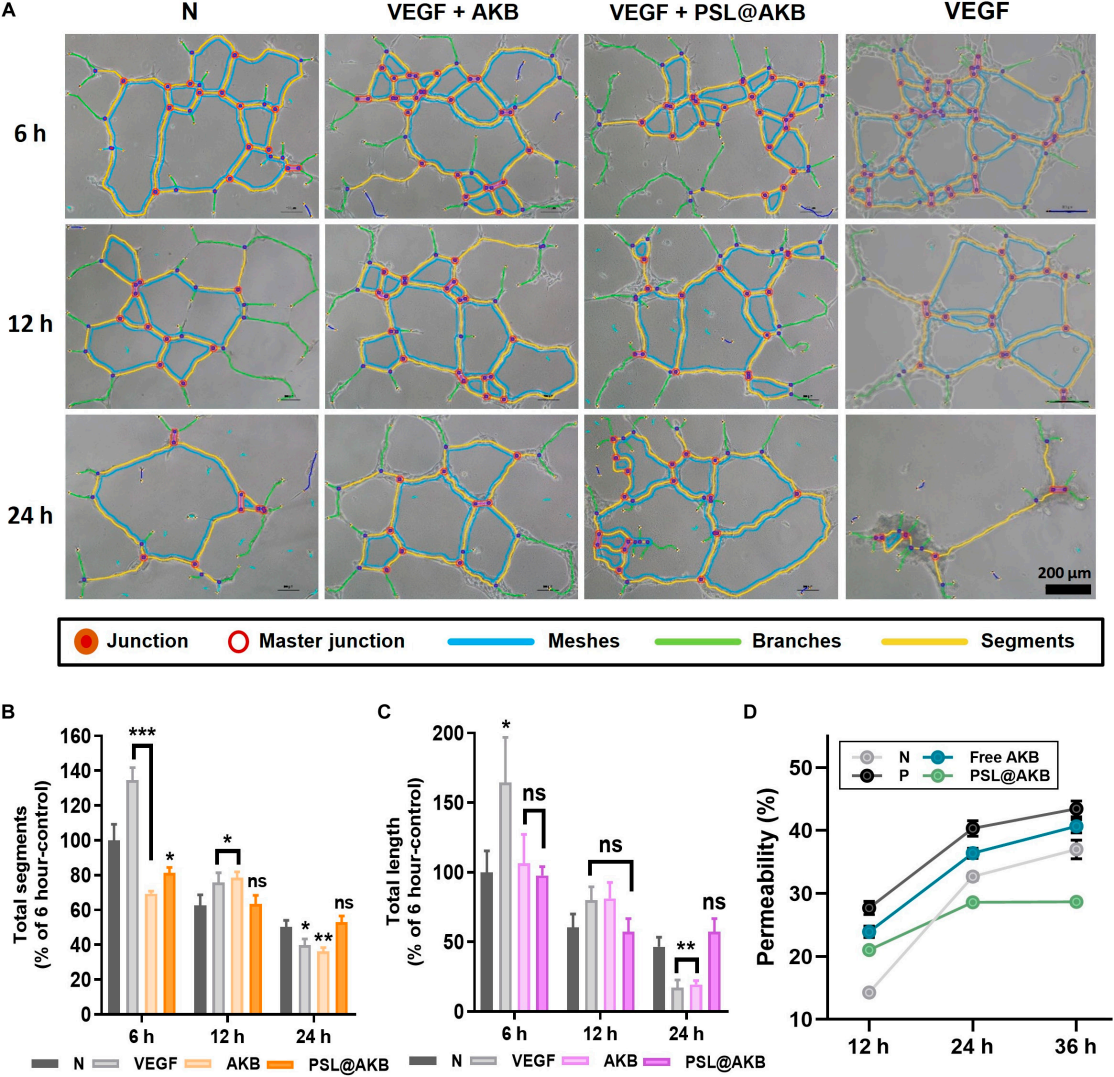
Stabilization of tube formation and permeability of HUVECs. (A) Tube formation assays were conducted using Matrigel, and bright-field images acquired at 6, 12, and 24 h were processed using ImageJ software (N, nontreated; P, treated with 10 ng/ml VEGF). (B) The total segment was graphed. (C) The total length was graphed. (D) Permeability test using a transwell system. FITC–dextran migration from the transwell chamber to the bottom plate well was quantified using a microplate reader at 12, 24, and 36 h (excitation: 488 nm, emission: 520 nm). Nonsignificant values have been represented as ns, while *, **, ***, and **** indicate *P* values < 0.0332, 0.0021, 0.0002, and 0.0001, respectively.

To evaluate angiogenesis in the vascular endothelial cells, a tube formation assay was performed using Matrigel. In general, when vascular endothelial cells are seeded on Matrigel, tube formation is most active between 8 and 16 h and is unstable after 24 h, characterized by a decrease in tube length and area [45]. We sought to induce an angiogenic environment so that tube formation proceeds more rapidly after treatment with VEGF, a pro-angiogenic factor. In this angiogenesis-inducing environment, we evaluated the effect of PSL@AKB treatment on tube stability following AKB-9778 delivery. Six hours after seeding, tube formation was most active in the group treated with VEGF (10 ng/ml) compared to the negative control group treated with nothing (Fig. 5A). The ability to form tubes decreased over time under all conditions. Light microscopy images obtained at specific time points were analyzed using the “Angiogenesis Analyzer” analysis program in ImageJ to obtain tube total segments and total length values, which were graphed as follows [46]. (Fig. 5B and C). It can be seen that the positive control group treated with VEGF alone showed rapid angiogenesis initially, but after 24 h, the tube length decreased most significantly. This could be attributed to more rapid angiogenesis in the presence of increased amounts of VEGF, increased endothelial cell migration, and decreased cell–cell interactions. In contrast, cotreatment with PSL@AKB particles showed a similar behavior to that of the negative control group, despite the same excess of VEGF, with a more pronounced difference after 24 h. Tube formation in the treated group was more stable than that in the untreated group. Free AKB-9778 showed the same trend, indicating that PSL@AKB can effectively deliver the drug and induce vascular stabilization.

Increased permeability of the blood vessel barrier is a major characteristic of tumor blood vessels. This enables blood leakage and leads to abnormal blood flow, with increased pressure on stromal cells [5–7,47]. Controlling the high permeability of tumor vessels is important because it hinders the accumulation of drugs that need to be delivered to the tumor. We performed a transwell permeability test to assess the permeability of vascular endothelial cells. HUVECs were cultured in transwell inserts with a pore size of 0.4 μm, and the permeability was compared by measuring the amount of FITC–dextran that escaped from the insert into the lower chamber. After 12 h, the permeability of vascular endothelial cells showed the lowest permeability of 14.3% in the normal condition, while the positive group treated with VEGF (10 ng/ml) alone showed the highest permeability of 27.72%. The permeability in the AKB-9778- and PSL@AKB-treated condition was lower than the positive condition at 27.72% and 23.93%, respectively. After 24 h, the permeability tended to increase rapidly to 32.7% in the normal condition. On the other hand, in the condition treated with PSL@AKB after VEGF (10 ng/ml), the permeability was lower than the normal condition at 28.63%. This trend remained the same after 36 h. The normal condition showed a permeability of 37.01%, while the positive and AKB-9778-treated conditions remained highly permeable at 43.46% and 40.69%, respectively. However, the PSL@AKB-treated condition maintained a similar permeability of 28.69% (Fig. 5D). This suggests that PSL@AKB can induce the stability of vascular endothelial cells by slow release drugs over a long period of time, which in turn can effectively reduce the increased permeability of vascular endothelial cells.

### Restoration of VE-cadherin in vascular endothelial cells using PSL@AKB

**Fig. 6.**
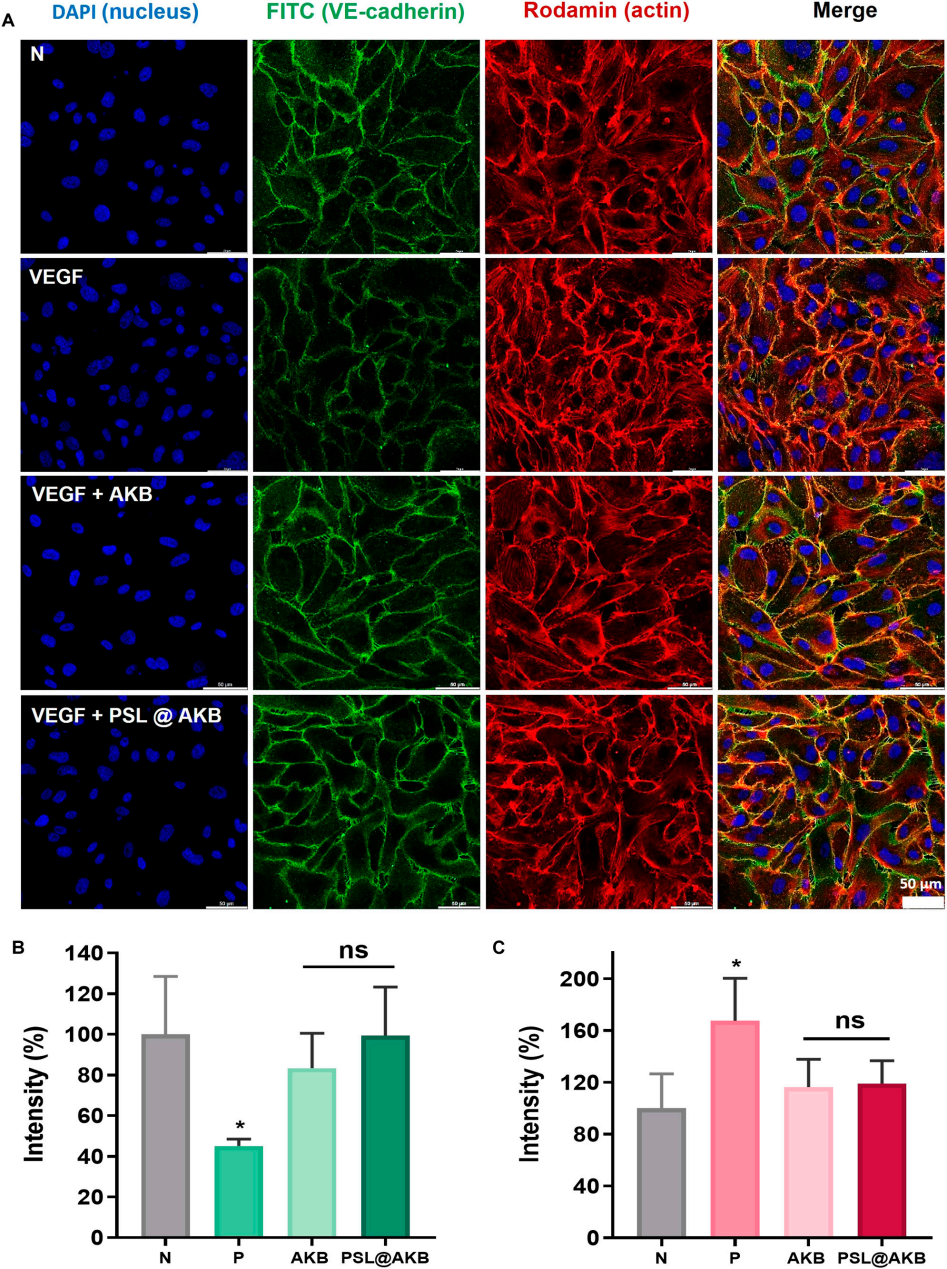
Restoration of VE-cadherin expression in vascular endothelial cells using PSL@AKB. (A) Confocal image. The expression levels of VE-cadherin and actin were compared using immunofluorescence in single-cultured HUVECs after treatment with VEGF (10 ng/ml) and treatment with free AKB and PSL@AKB. The expression level of VE-cadherin, which was attenuated by VEGF, was restored by treatment with PSL@AKB (HUVECs; blue, nucleus; green, VE-cadherin; red, actin). Analysis of fluorescence intensity of (B) VE-cadherin and (C) actin was performed using Leica Microsystems’ imaging software, LAS X, and the data are plotted as a graph. Nonsignificant values are represented as ns, while * indicate *P* values < 0.0001.

In the presence of high VEGF concentrations, PSL@AKB effectively induced tube stabilization in vascular endothelial cells and decreased vascular permeability. Normally, mature blood vessels have strong connections with vascular endothelial cells and maintain structural stability because of the adhesion proteins that connect them. We sought to determine whether there was a difference in adhesion protein expression in the stabilization of vascular endothelial cells induced by PSL@AKB delivery and used immunofluorescence to determine the expression levels of VE-cadherin, which belongs to the adherens junction. HUVECs cultured in complete medium uniformly expressed VE-cadherin on the junctional surface of neighboring cells. In contrast, treatment with VEGF (10 ng/ml) significantly decreased VE-cadherin expression levels and increased actin expression levels. In general, when angiogenesis occurs, the mobility of vascular endothelial cells increases; therefore, the force of binding proteins is weakened and the expression level of actin tends to increase as cells divide to create new blood vessels. When PSL@AKB was combined with VEGF, the expression level of VE-cadherin increased again, similar to the level in the negative control group, and the expression level of actin decreased. The results were similar when free AKB was administered; however, the expression of VE-cadherin was more effectively restored by PSL@AKB (Fig. 6A to C). Thus, PSL@AKB induced vascular endothelial cell stability by restoring the expression of weakened adhesion proteins, which can also regulate vascular permeability.

### Suppression of VEGF in a hypoxic HUVEC–cancer cell coculture system

**Fig. 7.**
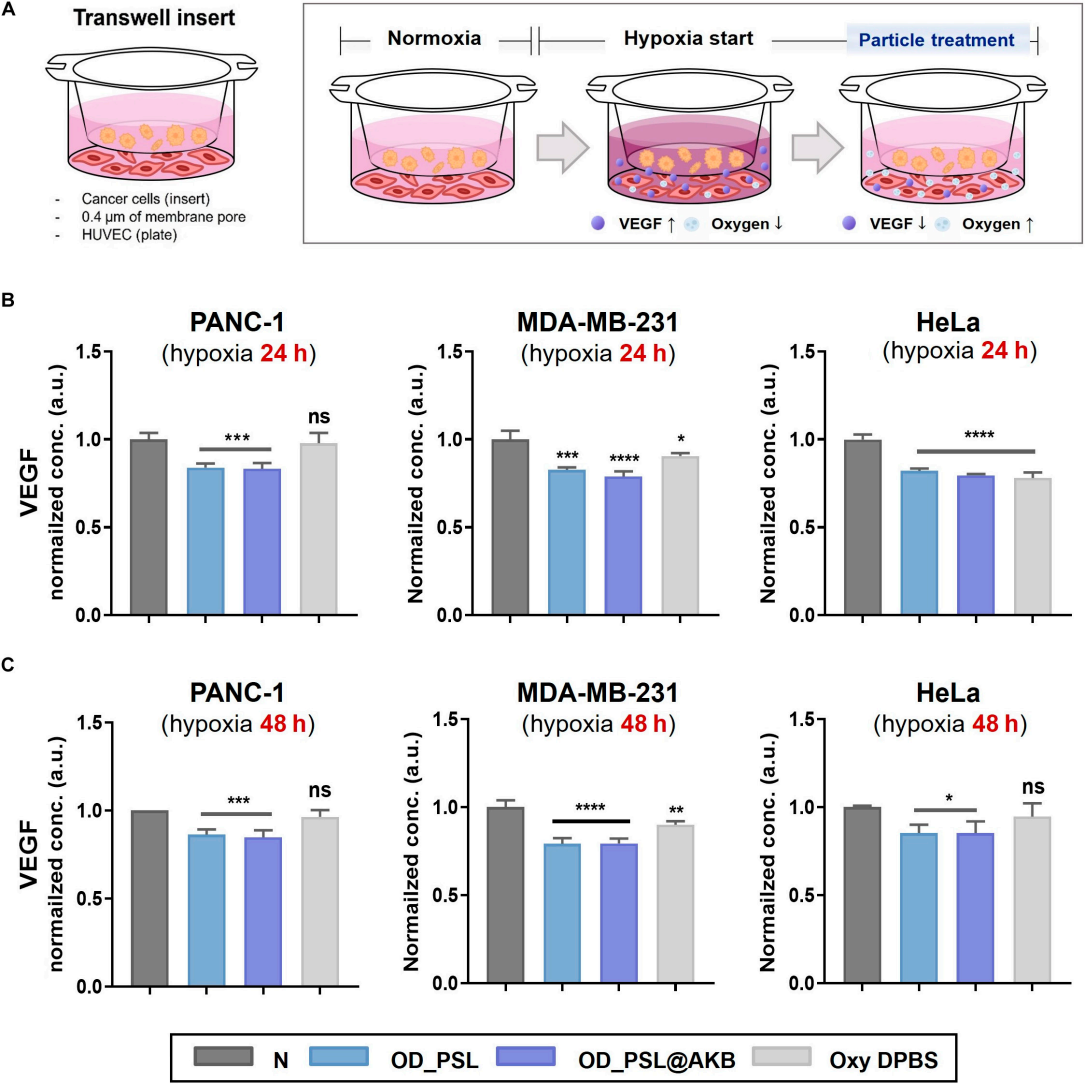
Suppression of VEGF in a hypoxic HUVEC/cancer coculture system. (A) Schematic representation of the entire experimental process of HUVEC/cancer cell coculture in hypoxia using a trans-well system. HUVEC/cancer cells (from left: PANC-1, MDA-MB-231, and HeLa) were cultured under hypoxia for (B) 24 h or (C) 48 h and treated with OD_PSL, OD_PSL@AKB, and Oxy-DPBS, and the changes in VEGF secretion were determined by ELISA. Nonsignificant values have been represented as ns, while *, **, ***, and **** indicate *P* values < 0.0332, 0.0021, 0.0002, and 0.0001, respectively.

Finally, we designed a transwell coculture system to coculture HUVECs and cancer cells to mimic peritumoral blood vessels. The cells were cultured in a hypoxic environment to induce VEGF secretion from the cancer cells (Fig. 7A). In general, cancer cells in hypoxic conditions increase the expression levels of the *HIF-1α* gene, which promotes the secretion of VEGF. Vascular endothelial cells stimulated by secreted VEGF show attenuated VE-cadherin expression. We evaluated whether the inhibition of VEGF expression and restoration of VE-cadherin could be achieved through delivery of the final particle, OD_PSL@AKB.

Various solid cancer cell lines, such as PANC-1 (pancreatic cancer), MDA-MB-231 (breast cancer), and HeLa (cervical cancer), were selected as the cancer cells and were cocultured with HUVECs in a hypoxic environment for 24 and 48 h. After treatment with particles and Oxy-DPBS for 4 h, the medium in the lower chamber was collected, and the concentration of VEGF was quantitatively analyzed using ELISA. The amount of VEGF secreted by the negative control group, without particle treatment, was used for normalization. After 24 h of hypoxia, treatment with the particles and Oxy-DPBS resulted in a decrease in VEGF secretion by all cancer cell lines, although there were differences in the extent of the decrease between the different cancer cell lines (Fig. 7B). VEGF secretion was reduced more by the particle treatment than by Oxy-DPBS treatment. Unlike Oxy-DPBS, which has no carrier to hold oxygen and is quickly released, OD_PSL and OD_PSL@AKB hold oxygen in the particles as carriers, indicating that oxygen can be supplied continuously. After 48 h of hypoxia, there was no significant difference in VEGF secretion in Oxy-DPBS; however, OD_PSL and OD_PSL@AKB effectively reduced VEGF secretion (Fig. 7C). This indicates that, even in an environment under hypoxia for a longer period, the particles can stably release oxygen to alleviate the hypoxic environment.

### Restoration of VE-cadherin expression by OD_PSL@AKB treatment in a hypoxic coculture environment

VEGF is one of the most potent pro-angiogenic factors that induce angiogenesis and are produced in excess under hypoxic conditions [48]. Hypoxia is highly correlated with tumor angiogenesis and the TME and promotes tumor growth and metastasis through immune evasion. Therefore, addressing the hypoxic environment is an important strategy in antitumor therapy. We successfully inhibited VEGF secretion from cancer cells under hypoxic conditions using OD_PSL@AKB, which is capable of delivering oxygen. This demonstrates the potential to relieve hypoxia in the TME and inhibit neoangiogenesis by reducing VEGF expression levels.

**Fig. 8.**
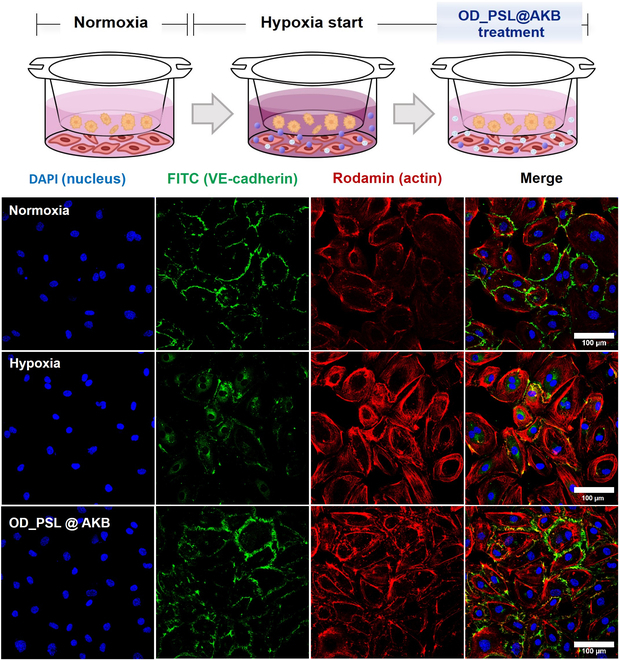
Restoration of VE-cadherin expression by OD_PSL@AKB treatment in a hypoxic coculture environment. VE-cadherin expression levels in HUVECs in a coculture environment were analyzed by immunofluorescence confocal microscopy. VE-cadherin expression in HUVECs was affected by coculture with cancer cells and was maximized in a hypoxic environment. The expression of VE-cadherin was then restored by OD_PSL@AKB treatment. This showed that OD_PSL@AKB can effectively achieve the recovery of VE-cadherin even in a hypoxic coculture environment.

We further investigated the expression of VE-cadherin in HUVECs using HUVEC/cancer cell cocultures under hypoxic conditions. We first compared the expression levels of VE-cadherin when HUVECs were monocultured in normoxic and hypoxic environments (Fig. S1). When HUVECs were cultured alone, there was no significant difference in VE-cadherin expression levels in either environment. We speculate that this was because there were no external factors, such as VEGF, that induce neovascularization. However, after coculturing with cancer cells, the expression level of VE-cadherin was reduced in the normoxic environment, and it was difficult to detect the expression of VE-cadherin in the hypoxic environment. In addition, the morphology of the HUVECs in the coculture changed compared with that in the monoculture. HUVECs that formed stable adhesion surfaces with neighboring cells were spaced apart, and the adhesion surfaces became pointed and elongated after coculture. The expression of VE-cadherin, which was greatly weakened by coculturing with cancer cells in this hypoxic environment, was restored after treatment with OD_PSL@AKB (Fig. 8). This indicates that structurally and functionally unstable tumor vessels can be normalized and induced to behave like normal vessels. These results suggested that OD_PSL@AKB is a promising co-delivery system that can relieve hypoxia through oxygen delivery, inhibit VEGF production, and normalize tumor vessels.

## Conclusion

Traditional anti-angiogenic therapy aims to inhibit tumor growth and metastasis by preventing the formation of blood vessels in tumors through the use of angiogenesis inhibitors. However, clinical studies have shown that while this method initially suppresses tumor growth, achieving complete tumor eradication is challenging, and drug resistance often arises over time. To address these issues, the concept of “vascular normalization” has been developed, which seeks to restore the structure and function of tumor blood vessels by balancing pro-angiogenic and anti-angiogenic factors. This strategy involves optimizing the dosage of anti-angiogenic inhibitors or targeting proangiogenic pathways. Despite its potential, vascular normalization still faces obstacles, including its limited duration of effectiveness and inadequate delivery of oxygen and drugs to the tumor [20,49,50]. In this study, we developed OD_PSL@AKB, which contains oxygen and AKB-9778, is sensitive to acidic pH, and can quickly release its cargo. It can selectively respond to an acidic TME to deliver oxygen and drugs and is expected to relieve hypoxia and normalize tumor vasculature. We found that the synthesized PSLs modified their particle morphology and shell structure when exposed to different pH environments, and we confirmed their increased drug release tendency in acidic environments (pH 5.5). We also evaluated whether these compounds could stabilize tube formation, reduce permeability, and restore weakened adhesion proteins in vascular endothelial cells. Finally, we compared the expression levels of VEGF and VE-cadherin in a hypoxic HUVEC/cancer cell coculture system. OD_PSL@AKB led to a decrease in VEGF by relieving hypoxia through oxygen delivery and effectively induced the stabilization of vascular endothelial cells by restoring the expression of VE-cadherin. We have shown that the OD_PSL@AKB platform not only promotes the normalization of unstable vascular structures but also efficiently mitigates the hypoxic conditions of the TME by directly delivering oxygen. It can also be used in combination with immunotherapy and chemotherapy to alleviate the hypoxic TME to promote an immune response or enhance the accumulation of drugs delivered to the tumor, leading to more positive results. In future studies, we plan to investigate the alterations in tumor vasculature and the activation of immune therapeutic responses after delivering OD_PSL@AKB in tumor-bearing small animal models. This will enable us to evaluate the effectiveness of its oxygen delivery and drug release properties under in vivo conditions and validate its potential for cancer treatment.

## Data Availability

The data that support the findings of this study are available from the corresponding author upon reasonable request.
