# Supplementary figures and images for: Lipid Nanoparticle Delivery System for Normalization of Tumor Microenvironment and Tumor Vascular Structure
Source: Biomater Res. 2025 Feb 11;29:0144. doi: 10.34133/bmr.0144 (PMC11811622; doi:10.34133/bmr.0144)

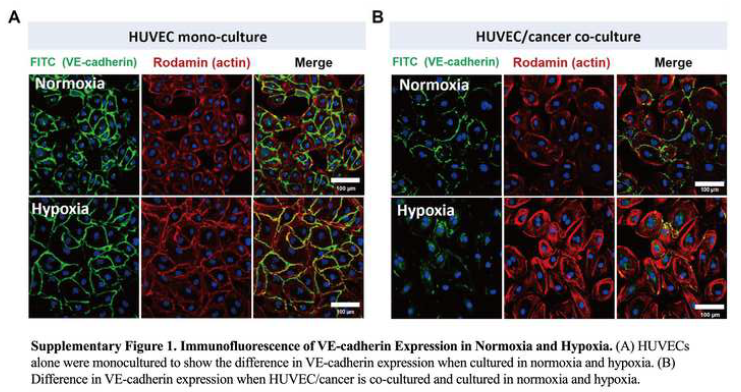

Supplement: Supplementary 1 — Fig. S1 [file bmr.0144.f1.docx]
